# Temperature-dependent development, predation, and life table of *Sphaerophoria macrogaster* (Thomson) (Diptera: Syrphidae) feeding on *Myzus persicae* (Sulzer) (Homoptera: Aphididae)

**DOI:** 10.1515/biol-2025-1183

**Published:** 2025-10-22

**Authors:** Jijun Fan, Lin Tan, Yishuai Yang, Wei Liu, Jihui Tan, Yong Li, Youcheng Miao, Qiulong Hu, Linghong Zhou

**Affiliations:** Chenzhou Institute of Agricultural Sciences, Chenzhou, 423000, Hunan, China; Hunan Agricultural University, Changsha, 410128, Hunan, China

**Keywords:** developmental rates, effective accumulative temperature, living habits, morphological characteristics, threshold temperature

## Abstract

*Sphaerophoria macrogaster* (Thomson) (Diptera: Syrphidae) is an important predator of *Myzus persicae* (Sulzer) (Homoptera: Aphididae). In this study, the morphological characteristics and living habits of *S. macrogaster* were observed and recorded. It evaluated the variations in developmental rate and predatory capacity of *S. macrogaster* when preying on *M. persicae* under five constant temperature regimes (16–32°C). Regression analyses assessing the relationship between temperature and the developmental rates of the egg, first, second, and third larval instars, and pupa of *S. macrogaster* were performed. In addition, the life table of hoverflies with *M. persicae* as prey at 28°C was determined. The results demonstrated that development rates at the egg, larval, and pupal stages gradually increased as the temperature increased from 16 to 32°C. The highest threshold temperature occurred at 7.93°C, in the egg stage. The threshold temperature and effective accumulated temperature of the whole generation of *S. macrogaster* were 5.56°C and 312.50° days, respectively. The larvae consumed more prey at 20°C than at other temperatures; however, the maximum value of the larva predation was 28.3 *M. persicae* per larva at 28°C, and the average duration of *S. macrogaster* development was 14.3 days from egg to adult emergence. The mortality of the egg stage of *S. macrogaster* was 18.0%, and the survival rate from the egg hatch to the adult stage was 58% at 28°C. These results provide valuable information for the release of *S. macrogaster* into managed systems to reduce *M. persicae* infestations under a wide range of temperature conditions.

## 1 Introduction

The green peach aphid, *Myzus persicae*, is one of the important tobacco (*Nicotiana tabacum* (L.) [Solanales: Solanaceae]) pests in China and around the world. As a highly polyphagous insect pest, *M. persicae* boasts an extremely broad host range, inflicting damage on over 40 plant families [1–5]. In spring, after tobacco is cultivated, *M. persicae* migrates into tobacco fields from other winter hosts (e.g., peach, cabbage, or sweet pepper). *M. persicae* damages tobacco plants by ingesting phloem, producing honeydew, and transmitting viral diseases (especially tobacco mosaic virus), causing curling and twisting of tender shoots, inducing the growth of black sooty molds, and resulting in the general devitalization of plants [6]. These damages decreased tobacco yield and quality in various tobacco-producing regions of China [7]. Currently, the prevention and control of *M. persicae* depend on chemical pesticides [8,9]. However, problems related to the use of chemical pesticides (e.g., resistance, residues, and resurgence) [3,10], as well as their negative effects on non-target organisms (e.g., inhibiting the growth, reproduction, and behavior of animals; interfering with physiological processes such as plant photosynthesis, hindering the growth, and reproduction of microorganisms; and damaging their ecological functions) [11–13], must be urgently addressed. As the economic, environmental, and health costs of chemical pesticide use become clearer [16,17], it is important to find other methods of pest control [17]. Encouragingly, in some areas, conventional chemical controls are gradually being replaced by biological controls based on integrated pest management systems [18,19].

Aphidophagous hoverflies (Diptera: Syrphidae) have long been recognized as important natural aphid predators [14,15]. Within the Syrphidae family, the larval stages of species in the Syrphinae and Pipizinae subfamilies exhibit predominant zoophagous behavior, with predation on sternorrhynchous Hemiptera (including aphids) as their primary feeding habit [20]. These two subfamilies collectively account for nearly one-third of all syrphid species [21]. Notably, the predatory role is fulfilled exclusively by the larval stage, whereas adult hoverflies typically rely on nectar and pollen for nutrition [22]. These flies can be found almost everywhere except Antarctica [23]. Because of their high levels of predation, they can effectively reduce aphid abundance [24,25]. Female hoverflies exhibit high mobility, which enables them to disperse eggs over extensive areas [26], and locate aphid communities earlier in the season than other aphidophaga [27,28] and lay their eggs near or directly in aphid colonies [29]. A study by Kakutani et al. [30] revealed that *Sphaerophoria macrogaster* was the dominant species among aphidophagous hoverflies at Kyoto in Japan. Previous studies have reported on the pollination and feeding behavior [31,32], species abundance [33], life history [34], and morphological characteristics of adults [35] of *S. macrogaster*. However, detailed reports on their living habits, biological characteristics, and potential biocontrol methods of *S. macrogaster* have not yet been available.

The study of living habits and morphology is important for field identification and collection efficiency, identifying feeding costs, and assessing the preventive capacity of *S. macrogaster*. Temperature influences the development and predation of natural prey, and higher and lower temperatures could lead to better or worse growth and development of insects, respectively [36,37]. Therefore, it is necessary to study the effects of temperature on the development and predation of *S. macrogaster* to determine the optimal temperature range for feeding and biocontrol application. Additionally, to use *S. macrogaster* as a biological control agent against *M. persicae*, some life parameters must be evaluated, including development and predation rates. The most important aspect of this evaluation is life table studies, which will provide a systematic understanding of the development and survival of *S. macrogaster* [38,39]. One of the most important factors contributing to the success of biological control is the coordination between natural enemies and environment [37], where natural enemies adapt to environmental conditions in which pests thrive. Previous research indicated that the optimum growth temperature [40] and development temperature [41] of *M. persicae* were both 28°C. Therefore, it is necessary to study the population life table of *S. macrogaster* at 28°C to evaluate its ability to control *M. persicae*.

The objectives of this study were to (i) observe and record the biological and morphological characteristics of the entire life cycle of *S. macrogaster* (eggs, larvae, pupae, and adults), (ii) study the effect of temperature on the development and predation capacity of *S. macrogaster* feeding on *M. persicae*, and (iii) evaluate the life tables of *S. macrogaster* feeding on *M. persicae* at 28°C.

## 2 Materials and methods

### 2.1 Aphid colonies and syrphid

The *M. persicae* colony and *S. macrogaster* of various stages were obtained from October 2016 to October 2018 in the tobacco fields (28°18ʹN, 113°07ʹE) of Changsha, Hunan Province, China. The insects were reared in our laboratory at room temperature (ca. 25 ± 1°C). Tobacco leaves with *M. persicae* were cut off and brought back to the laboratory, and *M. persicae* were reared on tobacco plants grown in vermiculite. The third or fourth instar nymphs of *M. persicae* measuring 1.2–1.5 mm were used in this experiment.

The larvae of *S. macrogaster* were transferred using a wolf-hair fine-lining brush and placed in Petri dishes (9 cm in diameter) in which the internal surface was covered with moist filter paper, and were fed with *M. persicae* until pupation. Petri dishes were checked every 12 h to ensure that *M. persicae* were available for feeding. Adult *S. macrogaster* were fed in insect cages (30 × 30 × 30 cm^3^). Fresh pollen (a bouquet of flowers of the oilseed rape plant and absorbent cotton soaked with 10% honey water were used to provide nutrition. The tobacco leaves with *M. persicae* were placed in each cage to stimulate the oviposition. Leaves were checked every 12 h and leaves with *S. macrogaster* eggs were replaced. After breeding, the eggs were transferred to Petri dishes (9 cm in diameter, with 1 egg per dish), and the date of oviposition was marked on each Petri dish. All eggs and larvae used in the experiment were obtained by this method.


**Ethical approval:** The research related to animal use has been complied with all the relevant national regulations and institutional policies for the care and use of animals.

### 2.2 Morphology and living habits of *S. macrogaster*

The eggs, larvae, pupae, and adults of *S. macrogaster* were placed under a Soptop SZN71 stereomicroscope (optical magnification range: 6.7–90×) to observe their morphological characteristics, and the adults were photographed using a Nikon stereo microscope Nikon smz18. The living habits of *S. macrogaster* were observed under both laboratory and field conditions, and the behavior (foraging, mating, and oviposition) were evaluated.

### 2.3 Effects of temperature on the development and predation of *S. macrogaster*

The Petri dishes with *S. macrogaster* eggs were placed into the artificial climate chambers with five treatments at 16, 20, 24, 28, and 32°C, at 75% (±1%) RH, photoperiod L:D = 12:12, and fed daily with 150 third or fourth instar nymphs. The amount of *M. persicae* consumed was recorded every 24 h, after which each syrphid larva was moved into a new Petri dish and supplied with sufficient *M. persicae*. The daily predation amount and the developmental time of *S. macrogaster* were recorded. Four *S. macrogaster* eggs were placed in Petri dishes (9 cm in diameter, 1 egg per dish) at each temperature, and each treatment was repeated five times.

### 2.4 Life table study and analysis

The temperature selected to evaluate the ability of *S. macrogaster* to adapt to environmental conditions in which *M. persicae* thrive is 28°C [40,41]. One hundred *S. macrogaster* eggs were placed in Petri dishes (9 cm in diameter, 1 egg per dish) at 28°C, and the date of oviposition was marked on each Petri dish. Each *S. macrogaster* larva was supplied with approximately 100 third or fourth instar nymphs each day. The pupae were transferred to new Petri dishes at the end of the larval stage, and the emerged adults of *S. macrogaster* were transferred to the insect cages (30 × 30 × 30 cm^3^). Fresh pollen (a bouquet of flowers of the oilseed rape plant and absorbent cotton soaked with 10% honey water were used to provide nutrition. The tobacco leaves with *M. persicae* were placed in each cage to stimulate the oviposition. Leaves were checked every 12 h and leaves with *S. macrogaster* eggs were replaced. If the eggs did not hatch or the pupae did not emerge for an additional week, they were considered dead. All developmental stages from the egg to the start of the oviposition of the adult were evaluated every day, and the developmental period of each individual spent in each life stage was obtained. Based on the age-stage life table, data on the survivorship and longevity of *S. macrogaster* were analyzed [24,38]. The age-stage specific survival rate (*S*_*xj*_) and the age-specific survival rate (*lx*) were then calculated. The age-stage specific survival rate (*S*_*xj*_) represents the probability that a single egg will develop to age *x* and stage *j*. It was calculated using the following equation:

(1) \[{S}_{{xj}}=\frac{{n}_{xj}}{{n}_{01}},]\]

where *n*_01_ denotes the number of individuals used at the start of the life table study and *n*_*xj*_ refers to the number of individuals that survive to age *x* and stage *j*.

The age-specific survival rate (*lx*) is the probability that a newborn will survive to age *x*. It was calculated using the following equation:

(2) \[lx=\mathop{\sum }\limits_{j=1}^{\beta }{S}_{{xj}},]\]

where *β* stands for the number of stages.

Note that this methodology considers each instar (i.e., first, second, and third instar larva) as distinct “stages” of the syrphid life cycle in the same way that egg, pupa, and adult.

### 2.5 Statistical methodology

Data were subjected to Kologorov–Smirnov tests and Levene’s tests to assess the normality and homogeneity of the variance, respectively. The Kruskal–Wallis test was used to analyze differences in the developmental times of the egg, larval, and pupal stages under five constant temperature conditions. Mean values were statistically compared with the Mann–Whitney test at *P* < 0.05 since data were not normally distributed. Regression analyses on the relationship between developmental rates and temperature were performed for each life stage and total life cycle. The following linear regression equation

(3) \[V=(1/K)\times T-\frac{C}{T}]\]

and least-square regression were used to estimate *K* (the required heat units in degree-days, DD) and *C* (the minimum developmental thresholds, DT), where *V* is the developmental rate in days at temperature *T* (°C).

## 3 Results

### 3.1 Morphological characteristics and living habits of *S. macrogaster*

**Figure 1:**
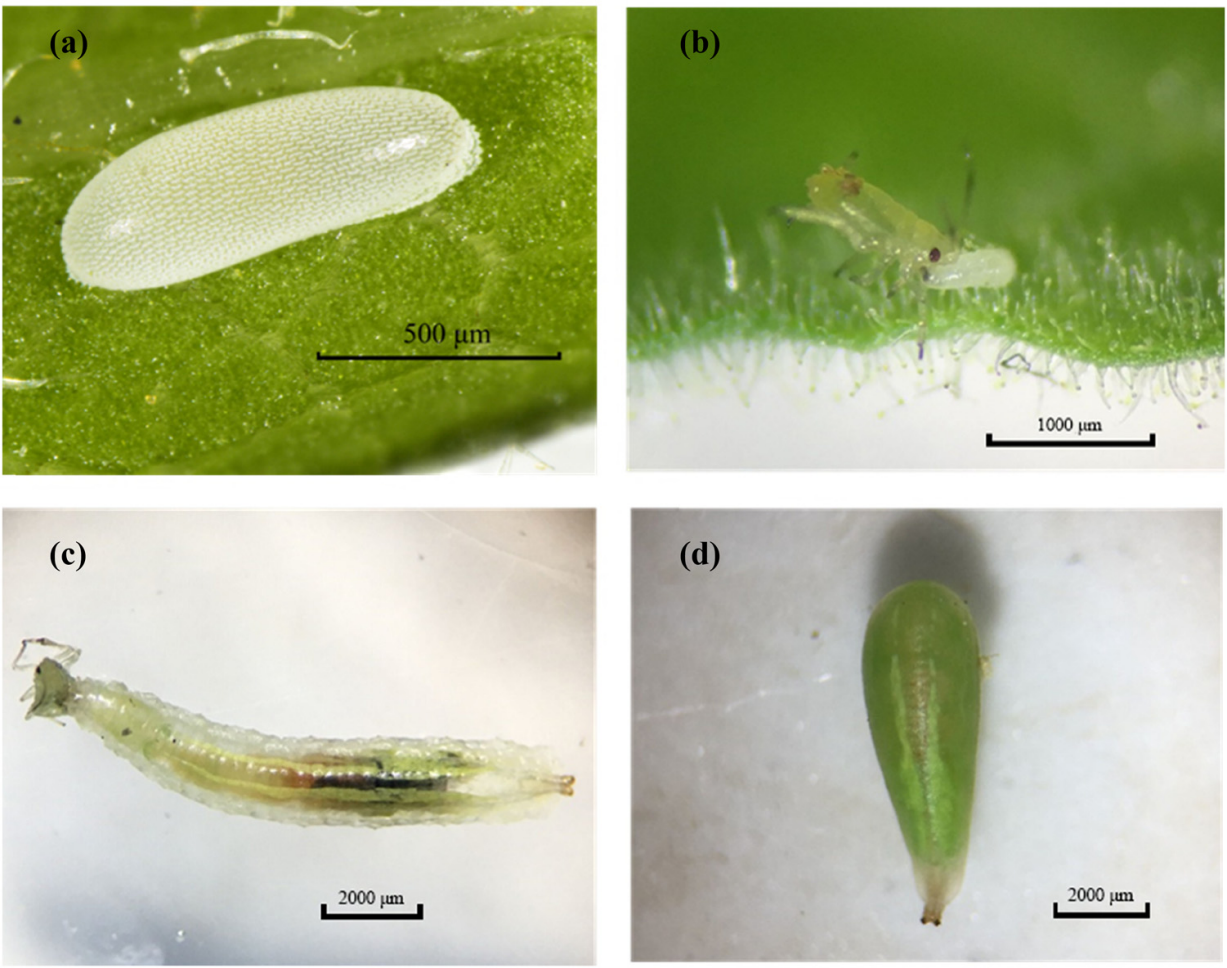
Morphological characteristics of egg (a), hatchling larvae (b), juvenile larvae (c), and pupae (d) of *S. macrogaster.*

**Figure 2:**
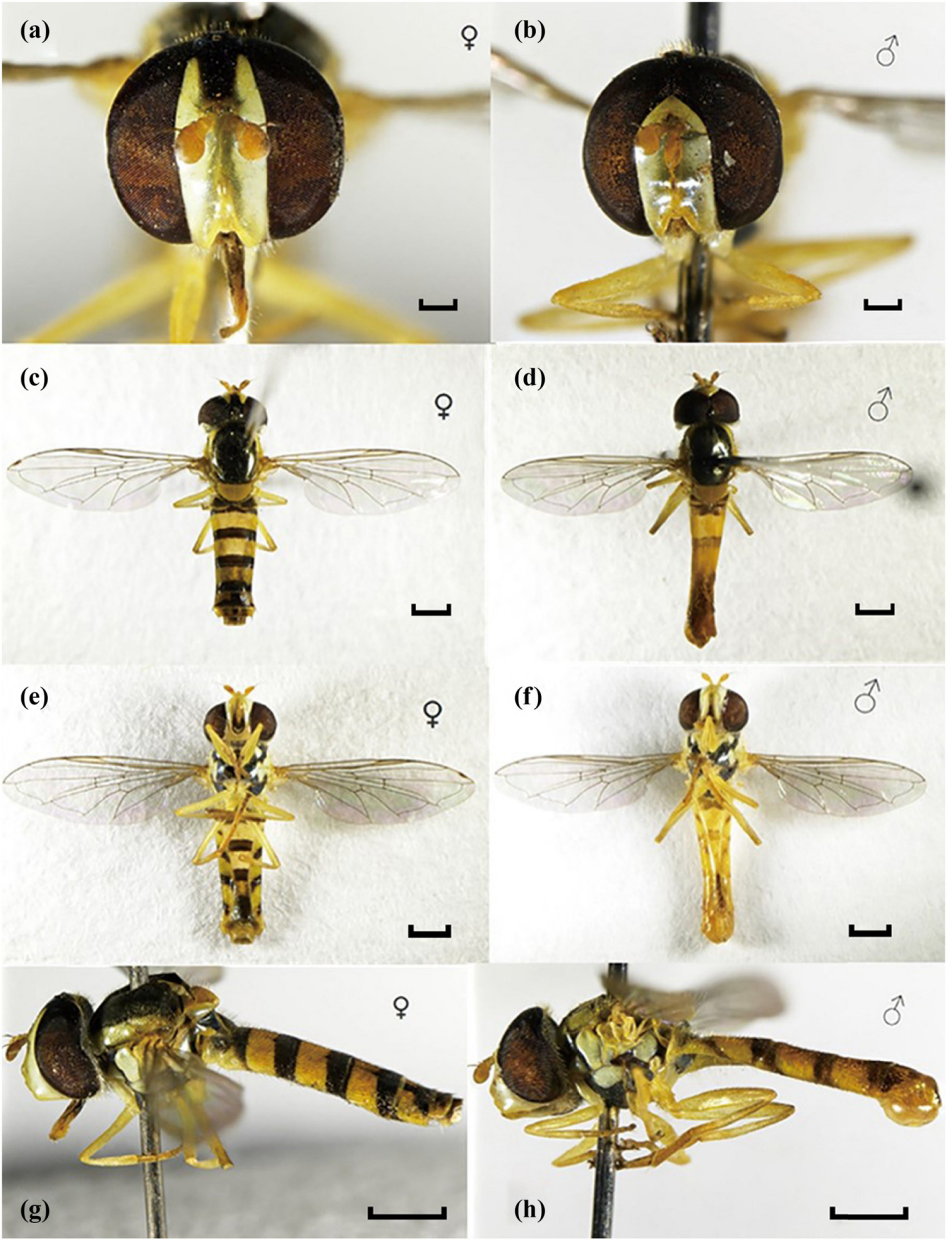
Morphological characteristics of adult of *S. macrogaster*. (a) female head, (b) male head, (c) female, dorsolateral view, (d) male, dorsolateral view, (e) female, ventral view, (f) male, ventral view, (g) female, lateral view, (h) male, lateral view.

*S. macrogaster* eggs are oval-shaped and a creamy white color, 0.7–0.9 mm long and 0.3–0.4 mm wide, with rough surfaces (Figure 1a). In nature, adult females of *S. macrogaster* typically lay eggs on the back of tobacco leaves or buds with *M. persicae*, and 1–3 eggs are usually distributed in each *M. persicae* population. However, under laboratory conditions, 13–24 eggs were observed on a tobacco leaf with *M. persicae*. It takes approximately 3–5 s to lay one egg. *S. macrogaster* larvae have three instars. First instar larvae are yellowish, 0.7–0.9 mm long. Second instar larvae gradually become translucent light green. The dorsum is characterized by two parallel white bands. Third instar larvae are 8.5–9.5 mm long and 1.5–1.8 mm wide, with a protuberant anus, and the two parallel strips on the dorsum change from white to light green. The newly hatched larva can feed on the adults of *M. persicae*, which takes over 20 min (Figure 1b), while third larval instars take 30 s to feed the adult aphids (Figure 1c). *S. macrogaster* adults are 5.9–7.2 mm long, with yellow antennae and legs. The black-yellow stripes are alternately arranged on the abdomen. Adult males and females can be distinguished by their eyes: male eyes are holoptic and female eyes are dichoptic (Figure 2). Different from larvae, adults of *S. macrogaster* must feed on pollen, nectar, or *M. persicae* honeydew to obtain nutrients and energy for their growth and development. Females must feed on pollen for their ovaries to mature. Both males and females mate multiple times throughout their lifetime, and the mating duration of adults is typically 3–8 min. A better understanding of the morphological characteristics and living habits of *S. macrogaster* provides support for the field identification and protection of this natural predator of *M. persicae*.

### 3.2 Effects of temperature on the developmental period of *S. macrogaster*

The mean developmental times of *S. macrogaster* in different stages at five constant temperatures are shown in Table 1. There were significant differences in the developmental durations at the egg, first, second, third larval instars, pupa, and egg to adult emergence stages of *S. macrogaster* under the following temperature conditions: 16, 20, and 24°C. However, at 28 and 32°C, only the developmental duration of pupae was significantly different. The developmental period for the egg, first, second, third larval instars, pupa, and egg–adult decreased as the temperature increased from 16 to 32°C. The average recorded duration of *S. macrogaster* development from egg to adult emergence was 29.8, 22.6, 16.6, 14.2, and 12.0 days at 16, 20, 24, 28, and 32°C, respectively.

**Table 1:** Mean (±SE) developmental time (days) of different stages of *S. macrogaster* at five constant temperatures

Temperature	Egg	Larval instars			Pupa	Egg–adult
		First	Second	Third		
16°C	4.6 ± 0.80a	4.2 ± 0.68a	4.0 ± 0.63a	7.2 ± 0.75a	9.8 ± 0.40a	29.8 ± 2.29a
20°C	2.8 ± 0.81b	3.2 ± 0.40b	2.8 ± 0.75b	5.6 ± 1.02b	8.2 ± 0.75b	22.6 ± 1.39b
24°C	2.0 ± 0.00c	2.2 ± 0.75c	1.8 ± 0.75c	4.4 ± 0.49c	6.2 ± 0.40c	16.6 ± 1.20c
28°C	1.8 ± 0.40c	2.0 ± 0.63cd	1.6 ± 0.49cd	3.6 ± 0.80d	5.2 ± 0.75c	14.2 ± 1.40d
32°C	1.6 ± 0.49c	1.6 ± 0.49d	1.6 ± 0.80d	3.4 ± 0.49d	3.8 ± 0.40d	12.0 ± 1.38d
*K*_4,95_	73.197	70.058	59.596	74.373	92.366	91.657
*P*	0.000	0.000	0.000	0.000	0.000	0.000

†Mean values in the same column with different letters differ significantly, *P*＜0.05.

### 3.3 Developmental rates of *S. macrogaster* at different temperatures

**Figure 3:**
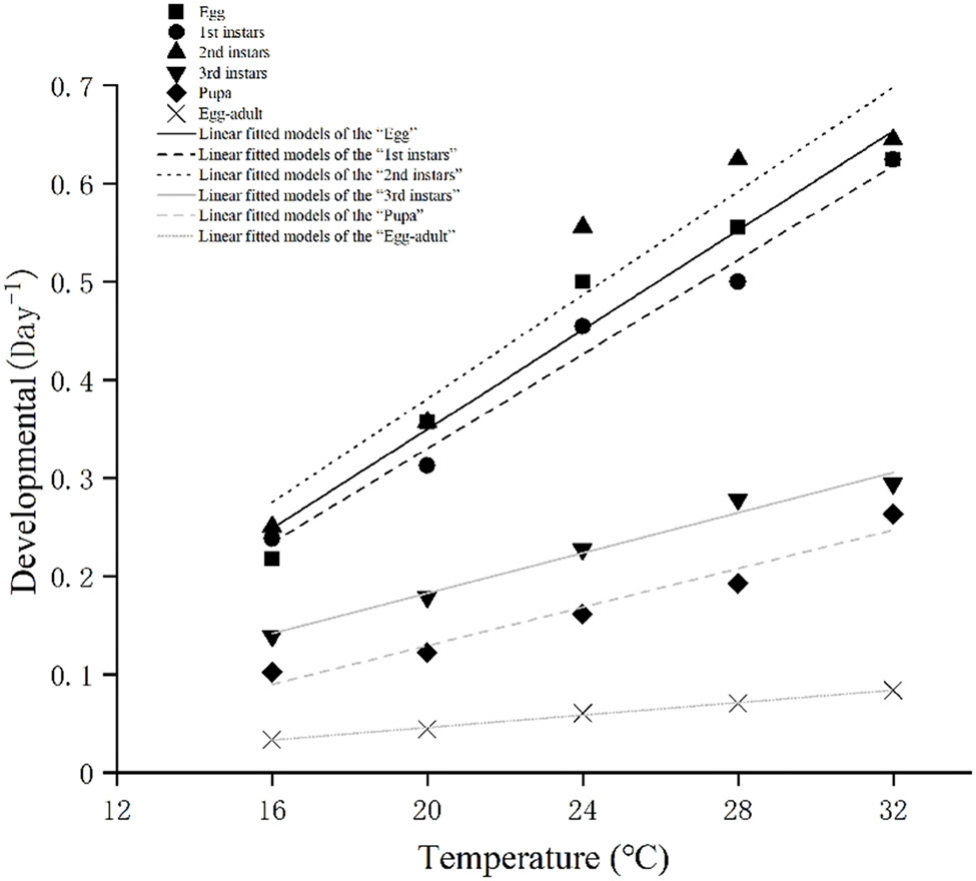
Linear fitted models of the development rate (1/development duration) for the total developmental time of *S. macrogaster*.

Lower temperature thresholds and degree-day requirements of *S. macrogaster* (Table 2) (*P* < 0.001) were calculated according to the linear regression equation (Figure 3), which was used to analyze the relationship between developmental rate and temperature. High coefficients of determination (*R* ^2^ > 0.9) were obtained for four stages: egg, third instar, pupa, and egg–adult. The highest threshold temperature for development occurred at 7.93°C in the egg stage. The threshold temperature for the developmental process of *S. macrogaster* from the egg to the adult stage was 5.56°C, and the effective accumulative temperature was 312.50° days (Table 2). According to the linear fitting model for the different developmental time of *S. macrogaster*, the developmental rate, sorted from high to low, are second instars > egg > first instars > third instars > pupa > egg–adult at each experiment temperature from 16 to 32°C (Figure 3).

**Table 2:** Linear regression analyses of relationship between developmental rates and temperature of different life stages of *S. macrogaster*

Stages	Regression equations	*R*²	*F* values	*P* > *F*	*C*	*K*
Egg	*V* = 0.0298*T* − 0.2363	0.9000	216.93	0.001	7.93	33.56
First instars	*V* = 0.0292*T* − 0.2283	0.8447	131.58	0.001	7.82	34.25
Second instars	*V* = 0.0338*T* − 0.2557	0.8536	140.96	0.001	7.57	29.59
Third instars	*V* = 0.0142*T* − 0.1117	0.9461	422.25	0.001	7.87	70.42
Pupa	*V* = 0.0101*T* − 0.0717	0.9178	268.88	0.001	7.10	99.01
Egg–adult	*V* = 0.0032*T* − 0.0178	0.9861	1699.19	0.001	5.56	312.50

†Calculated after Ding [55], where *T* is the temperature (°C), *V* is the developmental rate (1/developmental time), *K* is the effective accumulative temperature of insect degree-days, and *C* is the minimum developmental threshold (°C).

### 3.4 Effects of temperature on the predation amount of *S. macrogaster*

**Figure 4:**
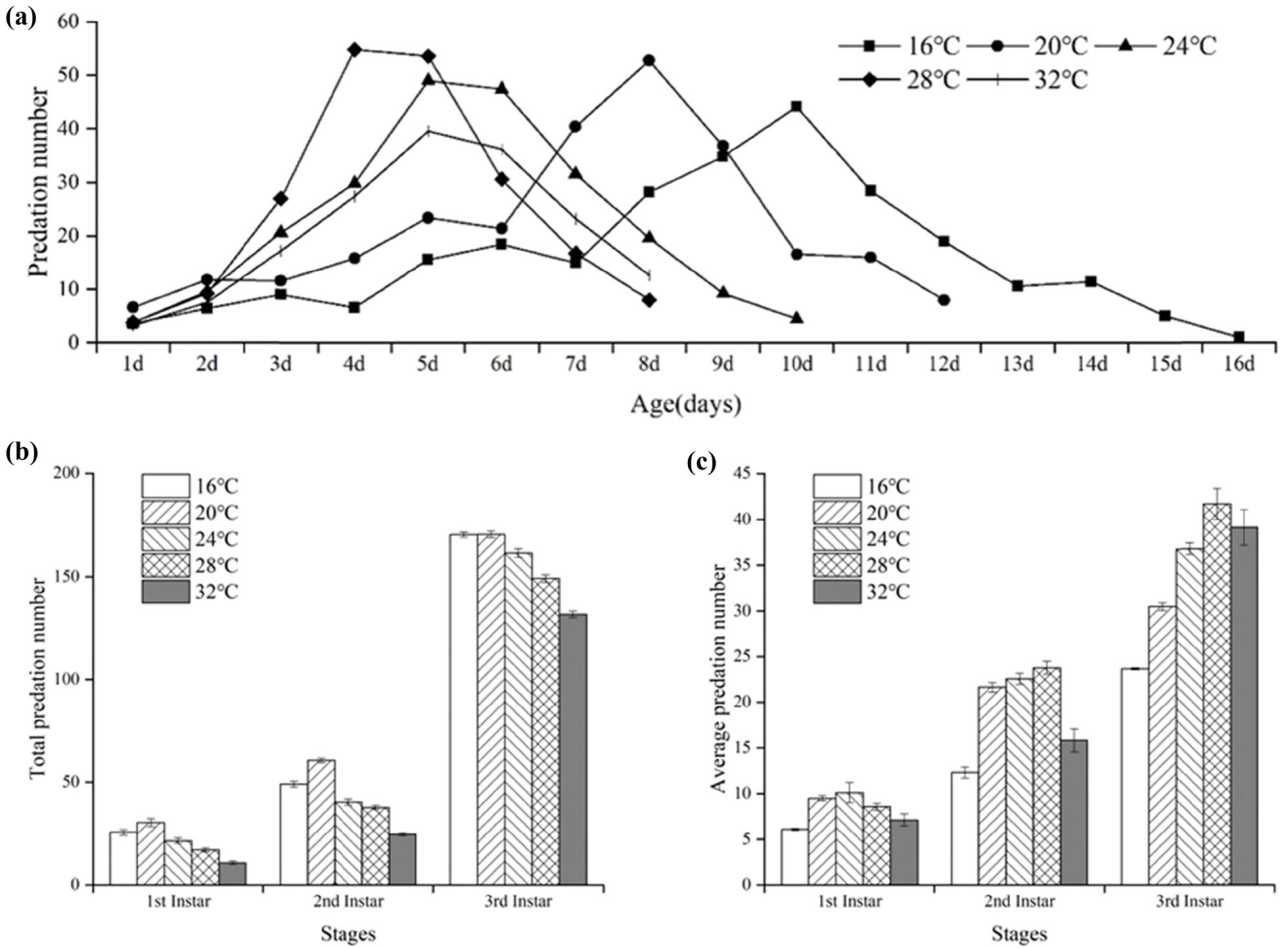
Daily predation amount (a), total predation amount (b), and average predation amount (c) of *S. macrogaster* on *M. persicae* at five constant temperatures.

The daily predation amount of *S. macrogaster* on *M. persicae* first increased and then decreased as temperature increased (Figure 4a). The shortest developmental durations (4 days) from the *S. macrogaster* larvae to the age of the highest daily predation amount was observed at 28°C, and the longest developmental durations (10 days) were observed at 16°C.

The effect of temperature on the predation amount of *S. macrogaster* larvae can be reflected by the difference in total predation amount (Figure 4b) and average predation amount (Figure 4c) at each developmental stage. The total predation amount and the average predation amount of *S. macrogaster* on *M. persicae* first increased and then decreased as temperature increased. The larvae consumed more prey at 20°C compared to other temperatures. The total predation amounts, sorted from high to low, are 261.2 *M. persicae* (20°C) > 245.0 *M. persicae* (16°C) > 225.1 *M. persicae* (24°C) > 203.8 *M. persicae* (28°C) > 167.0 *M. persicae* (32°C) (Figure 4b). The minimum predation amount for each developmental stage was observed at 16°C. The maximum value of the predation amount of the second and third larval instars and the total larval stages were all observed at 28°C. However, in the first instar, the maximum predation amount was observed at 24°C. At 28°C, the average predation amount of the total larval stages of *S. macrogaster* was 28.3 *M. persicae*/day (Figure 4c).

### 3.5 Life table of *S. macrogaster* at 28°C

The mean developmental time of *S. macrogaster* at different stages at 28°C is shown in Table 3. The successful hatching and emergence rates were 82 and 58%, respectively. Ultimately, 24 males and 34 females successfully emerged, for a sex ratio of 1:1.42. The average duration of *S. macrogaster* development was 1.9, 7.2, and 5.0 days for eggs, larvae, and pupae, respectively, and 14.3 days from egg to adult emergence.

**Table 3:** Mean developmental time (days) of different stages of *S. macrogaster*

Parameter	Stage	Mean ± SE	Percentage
Preadult	Egg	1.9 ± 0.35	100
	First instars	2.0 ± 0.43	82
	Second instars	1.8 ± 0.39	76
	Third instars	3.6 ± 0.79	74
	Larva (L1–L3)	7.2 ± 0.76	66
	Pupa	5.0 ± 0.71	66
	Egg–adult	14.3 ± 1.18	58
Adult	Female	11.3 ± 6.57	34
	Male	10.4 ± 6.00	24

†Percentage is the number of individuals observed at a certain stage, shows the probability of successful development to a certain stage.

**Figure 5:**
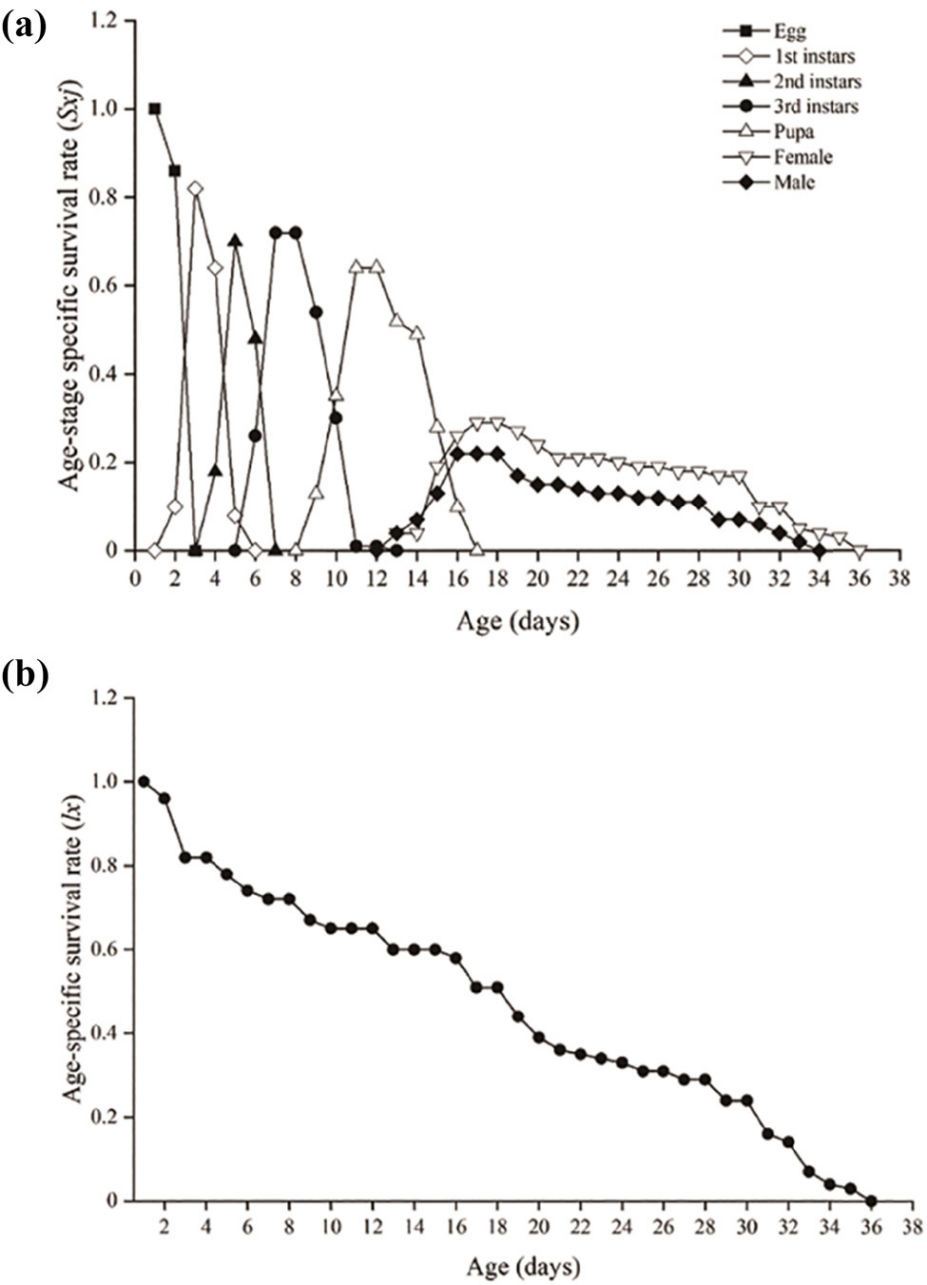
Age-stage specific survival rate (*S*_*xj*_) for each stage (a) and age-specific survival rate (*lx*) for the cohort (b) of *S. macrogaster* juvenile larva at 28°C and 75% RH.

The probability that a newly laid egg will develop to stage *j* and age *x* was analyzed by the age-stage specific survival rate (*S*_*xj*_) (Figure 5a). The survival rate of *S. macrogaster* from the egg hatch to the larval stage was 82% on the third day. The overlaps in the curves *S*_*xj*_ show the variable developmental rates among individuals of *S. macrogaster*. By ignoring the stage differentiation, the age-specific survival rate (*lx*) showed the survival percentage from the egg stage to age *x* (Figure 5b). The mortality rate of *S. macrogaster* was 18, 7, 3, 11, and 12% for eggs, first, second, third larval instars, and pupae, respectively. The probability of survival from the egg hatch to the adult stage is 58%, while the survival rate of *S. macrogaster* rapidly decreased at the early adult stage.

## 4 Discussion

The adults of syrphid are important pollinators in natural ecosystems and the larvae are well-known aphid predators and play an important role in suppressing aphids [24,25,42]. The species identification of syrphids and the morphological characteristics of adults have been described [43,44], but few studies have assessed the eggs and larvae. Tian and Ren [45] briefly summarized the morphological characteristics of larval and adult syrphids, but detailed characteristics of various syrphids were not assessed. Zhang et al. [46] described the morphological characteristics of the egg, larva, pupa, and adult of *Scaeva pyrastri* (Linnaeus) (Diptera: Syrphidae) and *Episyrphus balteatus* (De Geer) (Diptera: Syrphidae). Yu et al. [34] reported the biological characteristics of *S. macrogaster* and pointed out that the first instar larvae could not feed on adult *M. persicae*. In this study, the morphological characteristics and living habits of *S. macrogaster* were studied at each life stage, and we found that newly hatched *S. macrogaster* larvae could feed on adult *M. persicae*. Temperature is an important factor in the establishment and growth of insect populations [47]. Many studies have also shown that the effect of predatory hoverflies on aphids is closely related to temperature [48,49]. This study demonstrated that the developmental period for eggs, first, second, third larval instars, pupa, and egg–adults decreased as the temperature increased from 16 to 32°C, and the rates of development at each life stage gradually increased as the temperature increased from 16 to 32°C. These results were consistent with the results of previous studies, which found assessed the effect of temperature on the growth and development of hoverflies [50–52]. *M. persicae* typically harms tobacco in the summer [1], when high temperatures are conducive to the rapid reproduction of the *S. macrogaster*. This also creates favorable conditions for controlling *M. persicae*. Notably, across all tested temperatures, the developmental duration of the second larval instar was shorter than that of the first larval instar. These results were consistent with the results of previous studies. Singh et al. [25] reported that the durations of the first and second instars lasted 4.2 and 3.4 days for *Eupeodes frequens* (Matsmura) (Diptera: Syrphidae) and 4.8 and 3.3 days for *E. balteatus*, respectively; Arcaya et al. [24] reported that the durations of the both instars lasted 2 days for *Allograpta exotica* (Wiedemann) (Diptera: Syrphidae), respectively. In contrast, Faheem et al. [37] reported that the durations of the first and second instars lasted 2.67 and 4.70 days for *Ischiodon scutellaris* (Fabricius) (Diptera: Syrphidae) and 2.63 and 4.45 days for *E. balteatus*, respectively. This discrepancy may be related to species differences or experimental treatment conditions.

This study assesses *S. macrogaster* larvae feeding on *M. persicae* at different temperatures and provides information for future studies assessing whether temperature affects the ability of *S. macrogaster* to control *M. persicae* by releasing *S. macrogaster* into a greenhouse or field. The lower threshold for development and thermal constant are effective indicators for the establishment and growth of insect populations [50]. Our findings demonstrate that the threshold temperature and effective accumulated temperature of *S. macrogaster* from the egg to adult stage were 5.56°C and 312.50° days, respectively, indicating that *S. macrogaster* could survive in low-temperature environments. Daily predation levels of *S. macrogaster* on *M. persicae* at five constant temperatures first increased and then decreased as temperature increased. *S. macrogaster* had the highest predation ability at 28°C. Some studies have reported that the optimum growth temperature [40] and the fastest development temperature [38] of *M. persicae* to both be 28°C, which means that *S. macrogaster* could effectively control the *M. persicae* population during the peak reproductive period of *M. persicae*, though the predatory efficiency of *S. macrogaster* under fluctuating temperatures is not clear still. Our results suggest that the third instar larvae of *S. macrogaster* had the largest predation ability compared to first and second instar larvae, which is consistent with previous reports finding that hoverflies had the highest prey-consumption rates of syrphid flies as third instars [36,53,54]. The release of third instar larvae could be a more efficient control approach than the release of eggs or other instar larvae during *M. persicae* outbreaks. However, difficulties in the preservation and transportation of the third instar larvae remain a challenge for this approach.

To study the feasibility of *S. macrogaster* as a natural control against *M. persicae*, the life table of *S. macrogaster* from the egg stage to the adult’s first egglaying at 28°C was studied. Our results demonstrated that the average recorded duration of *S. macrogaster* development was 1.9, 7.2, and 5.0 days for eggs, larvae, and pupae, respectively, and 14.26 days from egg to adult emergence. Faheem et al. [37] reported that the average recorded duration of *I. scutellaris* development was approximately 3.8 and 6.7 days for eggs and pupae, respectively; the average recorded duration of *E. balteatus* development was approximately 3.1 and 7.7 days for eggs and pupae, respectively; and the average recorded durations of two species were approximately 11.0 days at 27°C. These lower developmental durations mean that the *S. macrogaster* could complete its life cycle faster than other syrphids. However, Dong et al. [47] reported that the average recorded duration for the development of *E. balteatus* was 1.80 and 6.08 days for eggs and pupae, respectively, at 27°C, which were lower than the results of Faheem et al. [37]. Arcaya et al. [24] reported a 34% mortality rate for *A. exotica* at the egg stage, but the mortality rate of the third instar stage of *A. exotica* was only 2%. Our results demonstrate that the mortality rate of *S. macrogaster* at the egg stage was 18%, and the mortality rate at the third instar stage was 11%. These phenomena may be ascribed to experimental rearing conditions, including excessively high temperatures or waterlogging induced by overly moist filter paper, or the direct collection of *M. persicae* to feed the larvae of *S. macrogaster*. In future studies, researchers should consider how to improve the survival rate of the third instar larvae.

This study was designed to investigate the morphological characteristics, living habits, the effect of temperature on the development and predation capacity, and life tables of *S. macrogaster* feeding on *M. persicae* at 28°C. The findings demonstrated that *S. macrogaster* exhibits sufficient environmental adaptability. These results are useful for rapidly identifying, commercially rearing, and optimizing the climatic conditions of *S. macrogaster*. However, its control effect against *M. persicae* requires further investigation.
